# Vinpocetine Attenuates the Osteoblastic Differentiation of Vascular Smooth Muscle Cells

**DOI:** 10.1371/journal.pone.0162295

**Published:** 2016-09-02

**Authors:** Yun-Yun Ma, Lin Sun, Xiu-Juan Chen, Na Wang, Peng-Fei Yi, Min Song, Bo Zhang, Yu-Zhong Wang, Qiu-Hua Liang

**Affiliations:** 1 Department of Endocrinology, Affiliated Hospital of Jining Medical University, Jining, Shandong Province, People’s Republic of China; 2 Jining Medical University, Jining, Shandong Province, People’s Republic of China; 3 Department of Neurology, Affiliated Hospital of Jining Medical University, Jining, Shandong Province, People’s Republic of China; 4 Central Laboratory, Affiliated Hospital of Jining Medical University, Jining, Shandong Province, People’s Republic of China; University of Sassari, ITALY

## Abstract

Vascular calcification is an active process of osteoblastic differentiation of vascular smooth muscle cells; however, its definite mechanism remains unknown. Vinpocetine, a derivative of the alkaloid vincamine, has been demonstrated to inhibit the high glucose-induced proliferation of vascular smooth muscle cells; however, it remains unknown whether vinpocetine can affect the osteoblastic differentiation of vascular smooth muscle cells. We hereby investigated the effect of vinpocetine on vascular calcification using a beta-glycerophosphate-induced cell model. Our results showed that vinpocetine significantly reduced the osteoblast-like phenotypes of vascular smooth muscle cells including ALP activity, osteocalcin, collagen type I, Runx2 and BMP-2 expression as well as the formation of mineralized nodule. Vinpocetine, binding to translocation protein, induced phosphorylation of extracellular signal-related kinase and Akt and thus inhibited the translocation of nuclear factor-kappa B into the nucleus. Silencing of translocator protein significantly attenuated the inhibitory effect of vinpocetine on osteoblastic differentiation of vascular smooth muscle cells. Taken together, vinpocetine may be a promising candidate for the clinical therapy of vascular calcification.

## Introduction

Vascular calcification is a common problem among the aged with diabetes, heart failure and end-stage renal disease [1]. It is correlated with a number of clinical complications such as myocardial infarction, impaired vascular tone, angioplasty dissection and poor surgical outcome [2]. Recent progresses suggest that vascular calcification is an active process in vascular smooth muscle cells (VSMCs) being similar to bone formation [3]. This process includes the expression of osteoblast-like phenotypes and the presence of the bone mineral hydroxyl apatite and matrix vesicles in VSMCs [3]. The definite mechanism of vascular calcification remains unknown by now. It is necessary to explore the mechanism of osteoblastic differentiation of VSMCs and develop effective strategies.

Vinpocetine, a derivative of the alkaloid vincamine, has been widely used in the treatment of cerebrovascular diseases and cognitive impairment for long time [4,5]. Generally, Vinpocetine significantly increased cerebral circulation and cerebral metabolism via a reduction in blood viscosity, the inhibition of Na^+^ channels and the scavenging of hydroxyl radicals [4]. As a inhibitor of phosphodiesterases, vinpocetine could modulate cholinergic functions, prevent neuronal cell damage through its antioxidant mechanism and thus improve spatial memory [6]. Recently, vinpocetine displays pleiotropic effect on multiple cell types and multiple physiological processes [7]. Typically, vinpocetine inhibited the high glucose-induced proliferation of VSMCs via preventing reactive oxygen species (ROS) activation and affecting MAPK, PI3K/Akt, and nuclear factor-kappa B (NF-кB) signaling [8]. Furthermore, vinpocetine attenuated neointimal hyperplasia and pathological vascular remodeling through suppressing ROS production [9]. However, it remains unknown whether vinpocetine could affect the osteoblastic differentiation of VSMCs. In this study, we presented the first evidences that vinpocetine significantly attenuated the beta-glycerophosphate (β-GP)-induced osteoblastic differentiation of VSMCs.

## Materials and Methods

### Reagents

Vinpocetine for injection was provided by Runhong Pharmaceuticals (Henan, China). Anti-mouse core binding factor a1 (cbfa1, Runx2), bone morphogenetic protein-2 (BMP-2), translocator protein (TSPO) and β-actin antibodies were purchased from Boisynthesis Biotechnology Company (Beijing, China). Anti-mouse ERK1/2, p-ERK1/2 (Thr202/Tyr204), p38, p-p38 (Thr180/Tyr182), c-Jun N-terminal kinases (JNK), p-JNK (Thr183/Tyr185), Akt, p-Akt (Ser473) and NF-кB p65 antibodies were purchased from Cell Signaling Technology Inc. (MA, USA). ERK inhibitor, PD98059 and Akt inhibitor, LY294002 were purchased from Calbiochem Corp. (San Diego, CA, USA). The alkaline phosphatase (ALP) assay kit was purchased from Nanjing Jiancheng Bioengineering Institute (Nanjing, China). The All-in-One First-Strand cDNA Synthesis Kit, All-in-One™ qPCR Mix and the primers for mouse Runx2, BMP-2, TSPO, ALP, collagen type I, osteocalcin, TGF-β1 and GAPDH were purchased from GeneCopoeia (Guangzhou, China).

### Vascular calcification *in vitro*

The primary mice VSMCs were prepared as our previously described [10]. Animal experiments were approved by the Ethics Committee of the Affiliated Hospital of Jining Medical University and performed in accordance with the Principles of Laboratory Animal Care. VSMCs were cultured in DMEM (Life Technologies Inc., NY, USA), containing 4.5g/l glucose, 10mM sodium pyruvate and 10% fetal bovine serum (FBS) (Life Technologies Inc., NY, USA) supplemented with 100U/ml of penicillin and 100μg/ml of streptomycin. VSMCs were passaged every 3–4 days, and experiments were performed between passages 3 and 8 from the primary culture. The cell model of vascular calcification was established in VSMCs using β-GP (10mM) as our previously reported [10].

### Effect of vinpocetine on β-GP-stimulated VSMCs

β-GP-stimulated VSMCs were respectively treated with 0, 5, 10, 15, 30 and 60 μM of vinpocetine. VSMCs without β-GP or vinpocetine were used as the control. After a culture of 48h, the cells were harvested for the apoptosis detection using an Annexin V-FITC/PI apoptosis detection kit (Beyotime, Jiangsu, China). Based on the apoptosis assay, we used not more than 15 μM of vinpocetine for the culture of VSMCs.

To explore the effect of vinpocetine on osteoblastic differentiation of VSMCs, β-GP-stimulated VSMCs were respectively treated with 5, 10 and 15 μM of vinpocetine for a culture of 7 days. VSMCs with no stimulus, β-GP-stimulated VSMCs treated without vinpocetine were used as the control. In another experiment, β-GP-stimulated VSMCs were treated with 15 μM of vinpocetine for 3, 6, 9 and 12 days respectively. After the culture, cells were harvested for the mRNA and protein expression of ALP activity, Runx2 and BMP-2.

To further confirm the effect of vinpocetine on calcifying VSMCs, VSMCs were seeded into a 24-well plate and cultured with β-GP or β-GP plus vinpocetine (15 μM). VSMCs without stimulus were used as the control. After a culture of 7 days, mRNA expression of collagen type I and osteocalcin were analyzed. After a culture of 18 days, Alizarin Red S staining was performed to detect the formation of mineralized nodules.

To explore the action mechanism of vinpocetine on osteoblastic differentiation of VSMCs, the β-GP-stimulated VSMCs were respectively treated with 15 μM of vinpocetine for 0, 5, 10, 15, 30 and 60 min. VSMCs treated with vinpocetine only were used as the control. After the incubation, the cells were harvested for the western blot analysis of expression of p38, ERK, JNK and Akt as well as their phosphorylation. Meanwhile, expression of NF-кB p65 in the nucleus was also analyzed.

To explore the relation between MAPK signaling and activation of NF-кB, PD98059 (10 μM), and LY294002 (10 μM) were respectively added into the VSMCs treated with β-GP and vinpocetine. After an incubation of 48h, the cells were harvested for the mRNA expression of Runx2 and BMP-2 as well as the ALP activity. The nucleus extracts were used for the NF-кB p65 expression. Alizarin Red S staining was performed to confirm the effects of PD98059 and LY294002 on calcifying VSMCs.

### Silencing of TSPO by RNA interference

TSPO, a 18 kDa protein consisting of 169 amino acids, has been demonstrated to be the specific receptor of vinpocetine [11]. To explore the relation between vinpocetine and TSPO in VSMCs, VSMCs with dose-gradient and time-gradient of vinpocetine were cultured for the analysis of TSPO. To explore whether vinpocetine affects the osteoblastic differentiation of VSMCs via TSPO, TSPO siRNA and the scramble siRNA (Gene Pharma Biotechnology, Shanghai, China) were transfected into VSMCs using Lipofectamine 2000 (Life Technologies Inc., NY, USA). After a culture of 48 h, the cells were harvested for the expression of TSPO, phosphorylation of ERK and Akt as well as the translocation of NF-кB p65 were detected by Western blot. Expression of Runx2, BMP-2 and TGF-β1 mRNA as well as ALP activity were also analyzed. After a culture of 18 days, Alizarin Red S staining was performed to detect the formation of mineralized nodules.

### Analysis of ALP activity

After the incubation at the indicated time, the cell layers were scraped into a solution containing 20 M Tris-HCl (pH 8.0), 150mM NaCl, 1% Triton X-100, 0.02% NaN_3_ and 1mM PMSF. The lysates were homogenized by sonication for 20s and assayed for ALP activity by measuring r-nitrophenol release at 37°C using a ChroMate® Microplate Reader (Awareness Technology, Palm City, USA) at 520 nm. Protein expression was normalized to total cellular protein by the Bradford protein assay.

### Measurement of mineralized matrix formation

After the incubation, cells were fixed in 95% ethanol for 10min at room temperature, washed with 2ml distilled water for three times, then stained with 1% Alizarin Red S for 1h at 37°C. After the staining, cell preparations were washed three times with distilled water to eliminate nonspecific staining. The formation of mineralized nodules was observed using bright filed in Axio Observer A1 inverted fluorescence microscope (Zeiss, Jena, Germany) and digital camera. The arizarin red S-positive area in the respective well was measured using Image J (version 6.0) (Media Cybernetics, Bethesda, MD).

### Western blot

Cytoplasmic protein and nuclear protein extracts of cultured cell were prepared using nuclear and cytoplasmic extraction lysate (Beyotime, Nantong, China). Equal amounts of protein were submitted to SDS-PAGE and transferred onto 0.2 μm PVDF membranes to be stained with appropriate antibodies (anti-p-p38, p38, p-ERK, ERK, p-JNK, JNK, Akt, p-Akt, NF-кB p65, Runx2, BMP-2, TSPO and β-actin). The reaction was visualized with chemiluminescence. The results were calculated using the software of Image J for windows by comparing the gray value (Area multiplied by mean gray value) between target protein and β-actin.

### Quantitative PCR

Total RNA was extracted by TRIzol (Life Technologies Inc., NY, USA), and then cDNA was prepared with an All-in-One™ First-Strand cDNA Synthesis Kit. Amplification and detection were performed in an ABI 7500 as follows: 95°C for 10min and then 40 cycles of 95°C for 10s, 60°C for 20s, and 72°C for 30s. A total 20μl of reaction system consists of SYBR Mix 10μl, Rnase-free water 5.6μl, 2μl forward/reverse primer (2μM), 2μl cDNA template and 0.4μl of ROX. GAPDH was used as the inner control. A semi-quantitative method employing 2^-ΔΔCt^ was used to analyze the mRNA expression as previously reported [12].

### Statistical analysis

Representative results from three independent experiments were shown and were presented as mean ± standard error. Differences between groups were evaluated by one-way analysis of variance. A level of p<0.05 was considered significant. Analysis was performed with the SPSS 20.0 analysis software by IBM (Armonk, NY, USA).

## Results

### Vinpocetine significantly inhibited the osteoblastic differentiation of VSMCs

**Fig 1:**
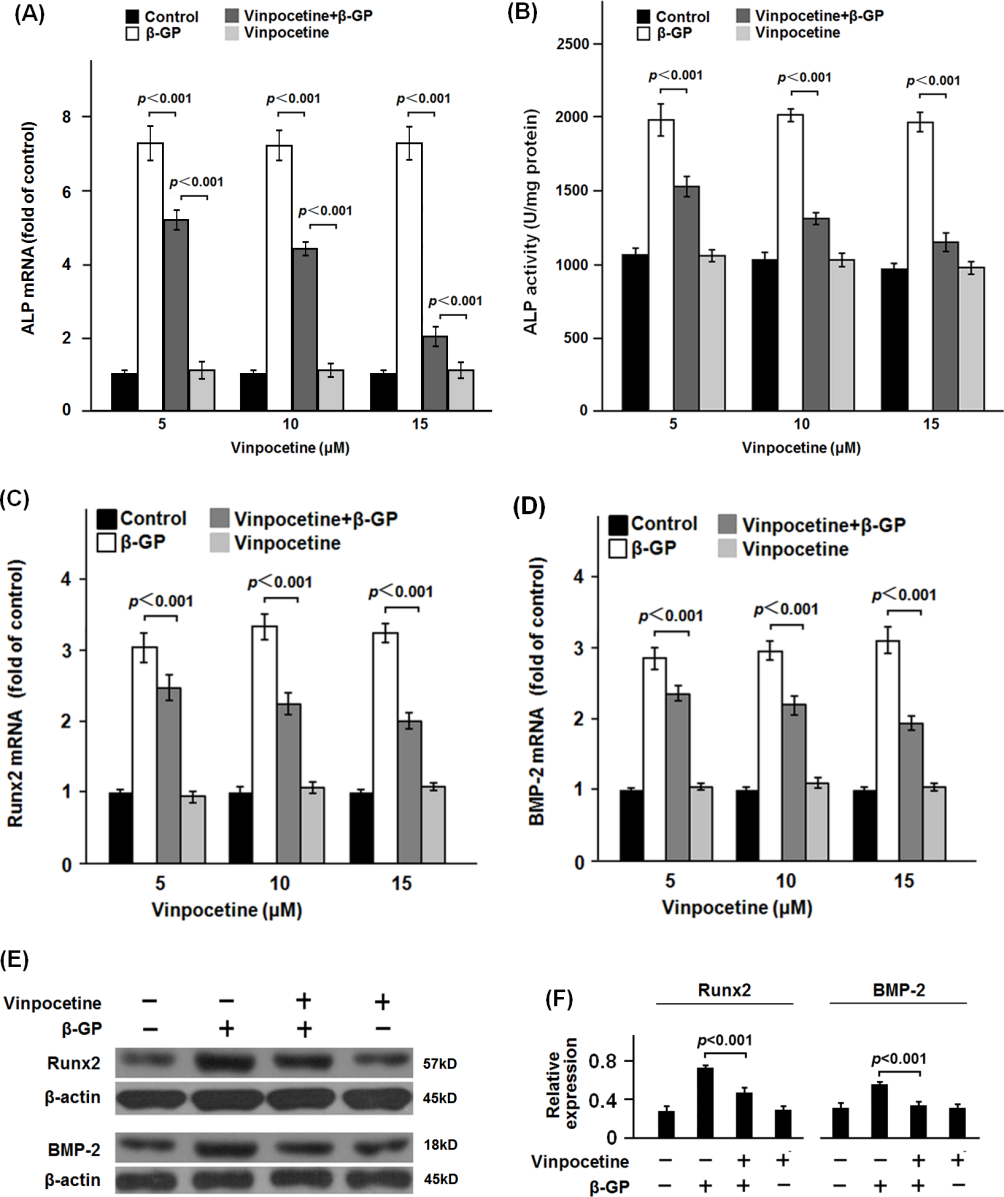
Effect of dose-gradient vinpocetine on ALP activity, Runx2 and BMP-2 expression in β-GP-stimulated VSMCs. VSMCs were stimulated with β-GP to establish the cell model of vascular calcification. Vinpocetine was added into the culture respectively at the indicated concentration for 7 days. After the culture, cells were harvested for the analysis of ALP (A and B), expression of Runx2 and BMP-2 (C-F). As it was shown, vinpocetine significantly inhibited the ALP activity and the expression of Runx2 and BMP-2 in β-GP-stimulated VSMCs. Representative results from three independent experiments were presented as mean ± standard error.

**Fig 2:**
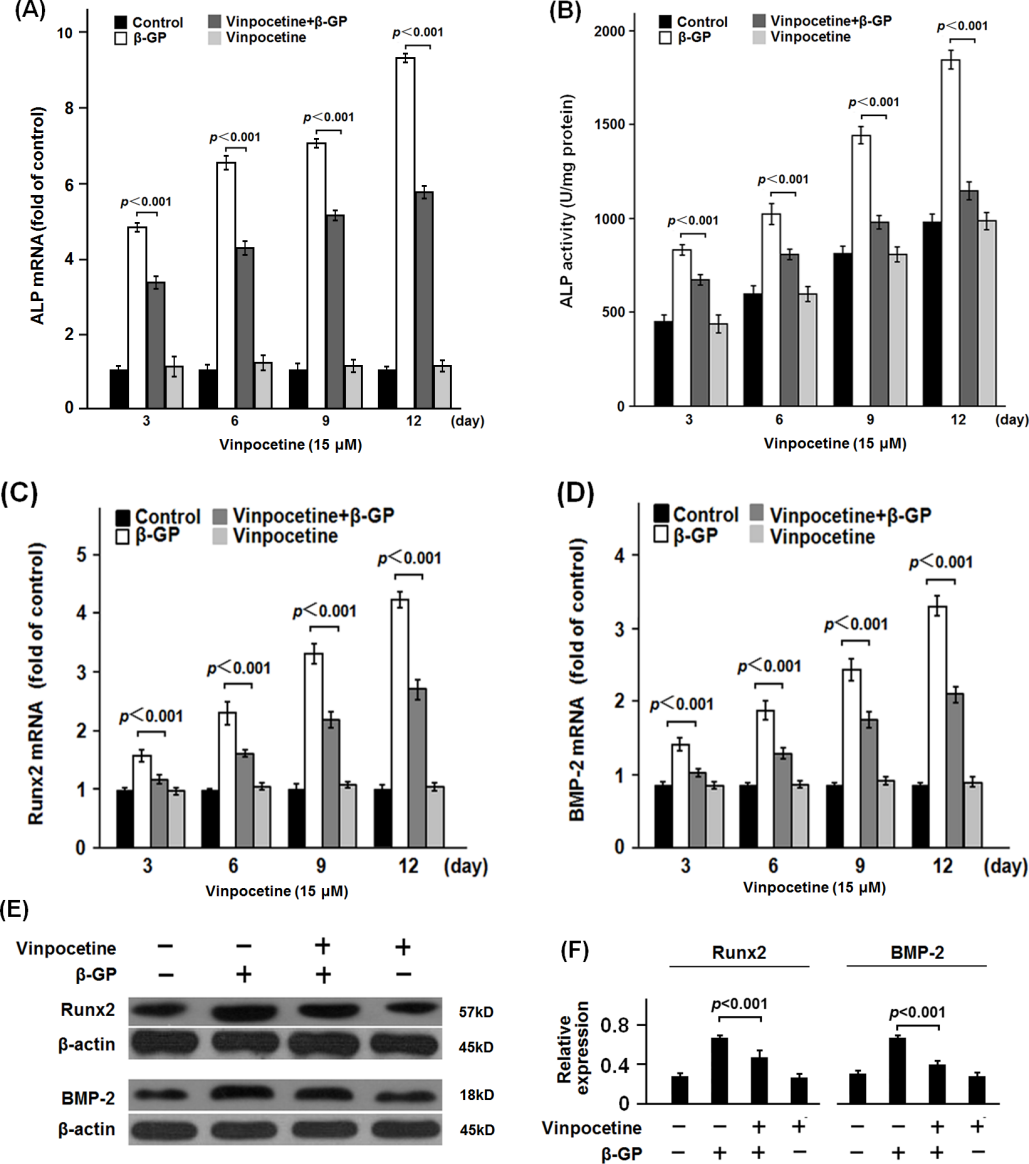
Effects of time-gradient vinpocetine on ALP activity, Runx2 and BMP-2 expression in β-GP-stimulated VSMCs. VSMCs were stimulated with β-GP to establish the cell model of vascular calcification. The cultured VSMCs were treated with or without 15μM of vinpocetine for the indicated time periods respectively. After the culture, cells were harvested for the analysis of ALP activity (A and B), expression of Runx2 and BMP-2 (C-F). As it was shown, vinpocetine significantly inhibited the ALP activity and the expression of Runx2 and BMP-2 in β-GP-stimulated VSMCs. Representative results from three independent experiments were presented as mean ± standard error.

**Fig 3:**
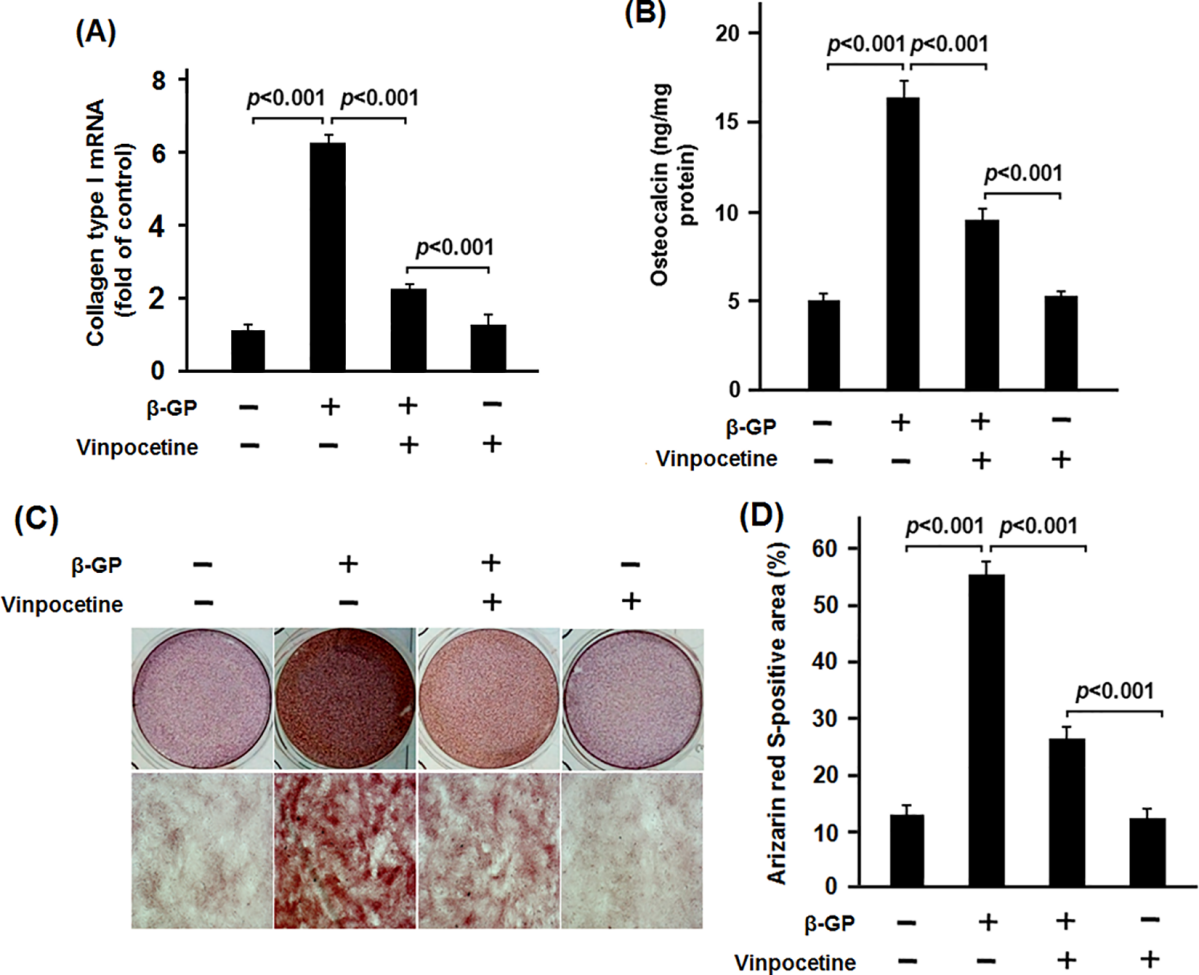
Vinpocetine reduced the expression of collagen type I and osteocalcin as well as the matrix mineralization of β-GP-stimulated VSMCs. β-GP-stimulated VSMCs with or without 15μM of vinpocetine were cultured for 7 days. The cells were harvested to extract the total RNA. qPCR was performed to detect the expression of collagen type I and osteocalcin. As it was shown (A-B), vinpocetine significantly reduced the mRNA expression of collagen type I and osteocalcin in β-GP-stimulated VSMCs. After a culture for 18days, the cells were stained using Alizarin Red S. There was significant less mineralized nodules formation in β-GP plus vinpocetine-treated VSMCs than the β-GP only (C-D). Representative results from three independent experiments were shown.

To explore the effect of vinpocetine on osteoblastic differentiation of VSMCs, VSMCs were treated with dose-gradient vinpocetine. The apoptosis assay showed that 30 μM or more of vinpocetine could induce significant apoptosis of β-GP-stimulated VSMCs (data not shown). ALP, Runx2 and BMP-2 are well established phenotypic markers of calcifying VSMCs [13,14]. In the dose-gradient experiment, 5 to 15μM of vinpocetine significantly inhibit the ALP activity and the mRNA expression of ALP (Fig 1A and 1B). 5 to 15μM of vinpocetine could significantly inhibit both the mRNA and protein expression of Runx2 and BMP-2 (Fig 1C–1F, the data of western bloting analysis of 5 and 10μM of vinpocetine on expression of Runx2 and BMP-2 were not shown). Similarly, in the time-gradient experiment, 15μM of vinpocetine significantly reduced the ALP activity and its mRNA expression in the β-GP-stimulated VSMCs from 3 to 12 days (Fig 2A and 2B). There was significant less expression of both the mRNA and protein of Runx2 and BMP-2 in VSMCs treated with β-GP and vinpocetine than those treated with β-GP only (Fig 2C–2F, the data of western bloting analysis of Runx2 and BMP-2 on day 3, 9 and 12 were not shown). Osteocalcin and collagen type I are critical for the osteoblastic differentiation of VSMCs [15]. Our data showed that vinpocetine significant reduced the mRNA expression of osteocalcin and collagen type I in β-GP-stimulated VSMCs (Fig 3A and 3B). As it was shown in Fig 3C and 3D, Alizarin Red S staining showed that 15 μM vinpocetine significantly reduced the formation of mineralized nodule in β-GP-stimulated VSMCs.

### Vinpocetine inhibited the osteoblastic differentiation of VSMCs via ERK/Akt signaling pathway

**Fig 4:**
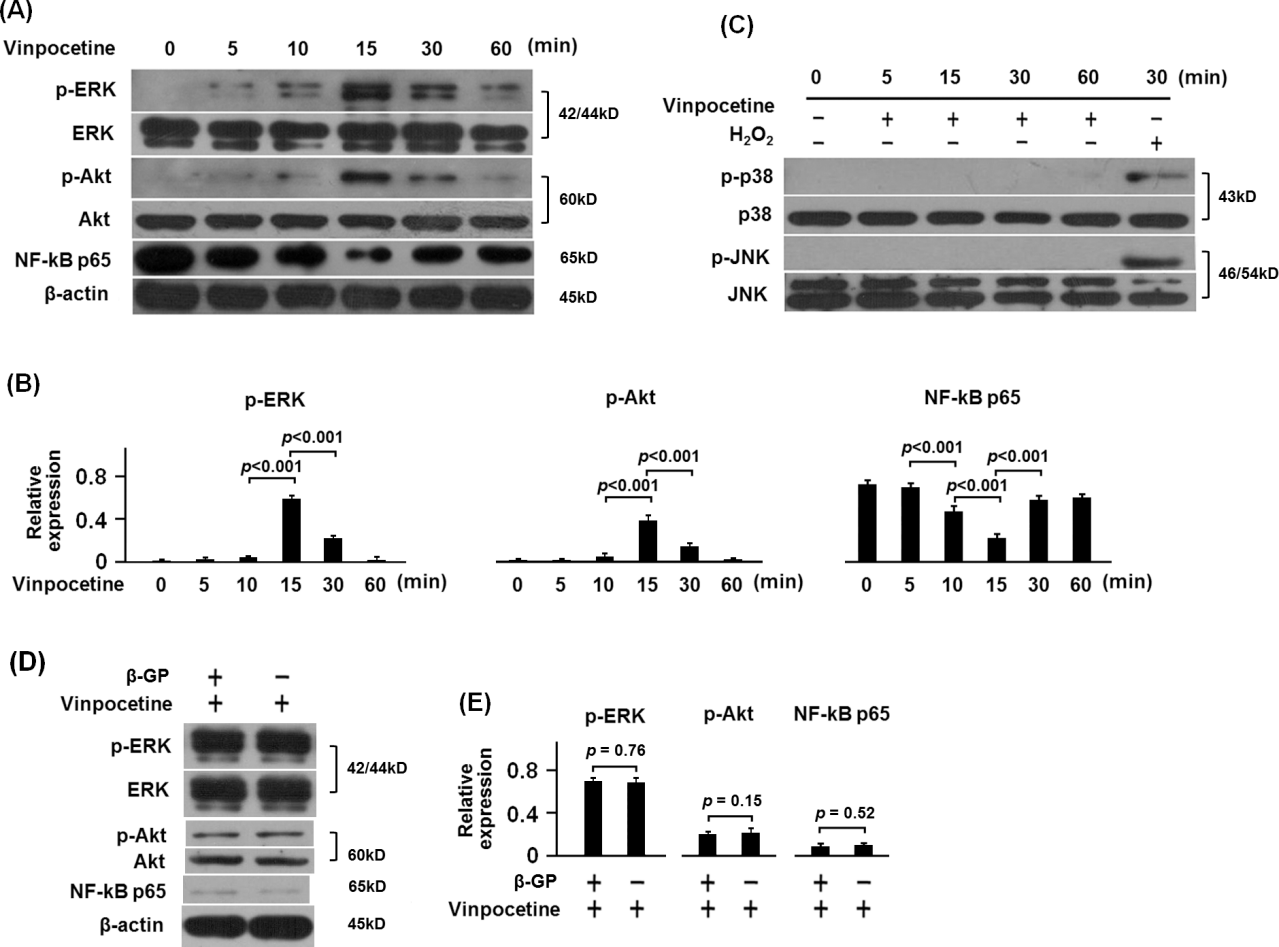
Effect of vinpocetine on phosphorylation of MAPK and Akt as well as the translocation of NF-кB p65 in β-GP-stimulated VSMCs. β-GP-stimulated VSMCs were treated with vinpocetine for the indicated time. After the culture, VSMCs were harvested for the Western blot analysis. As it was shown, 15μM of vinpocetine induced significant phosphorylation of ERK and Akt as well as the inhibition of NF-кB translocation in VSMCs at 5min after treatment, and the effect of vinpocetine peaked at 15min after treatment (A-B). Neither p38 nor JNK were phosphorylated by the treatment of vinpocetine (C). To further confirm the effect of vinpocetine on phosphorylation of ERK, Akt and translocation of NF-кB in VSMCs, VSMCs with and without β-GP were respectively treated with vinpocetine for 15 min. As it was shown (D-E), there was no difference in the phosphorylation of ERK and Akt as well as the expression of NF-кB p65 in the nucleus between VSMCs treated with β-GP plus vinpocetine and those with vinpocetine only. Representative results from three independent experiments were shown.

Mitogen-activated protein kinase (MAPK) and PI3K/Akt were well known to play an essential role in controlling cell differentiation. It has been shown that vinpocetine could inhibit the high glucose-induced proliferation of VSMCs by affecting MAPK, PI3K/Akt and NF-кB signaling [8]. To explore the action mechanism of vinpocetine on osteoblastic differentiation of VSMCs, we detected the phosphorylation of ERK, p38, JNK and Akt as well as the translocation of NF-кB p65 into the nucleus. Notably, vinpocetine treatment induced significant phosphorylation of ERK and Akt in β-GP-stimulated VSMCs, which peaked at the 15 min after the vinpocetine treatment. The vinpocetine significantly inhibited the translocation of NF-кB p65 and this inhibition also peaked at the 15 min after the vinpocetine treatment. The details were shown in Fig 4A, 4B and 4D. Neither JNK nor p38 were phosphorylated by the 15 μM vinpocetine treatment (Fig 4C). Hydrogenperoxide (H_2_O_2_) was used as the positive control to confirm the results from the analysis of the phosphorylation of JNK and p38.

**Fig 5:**
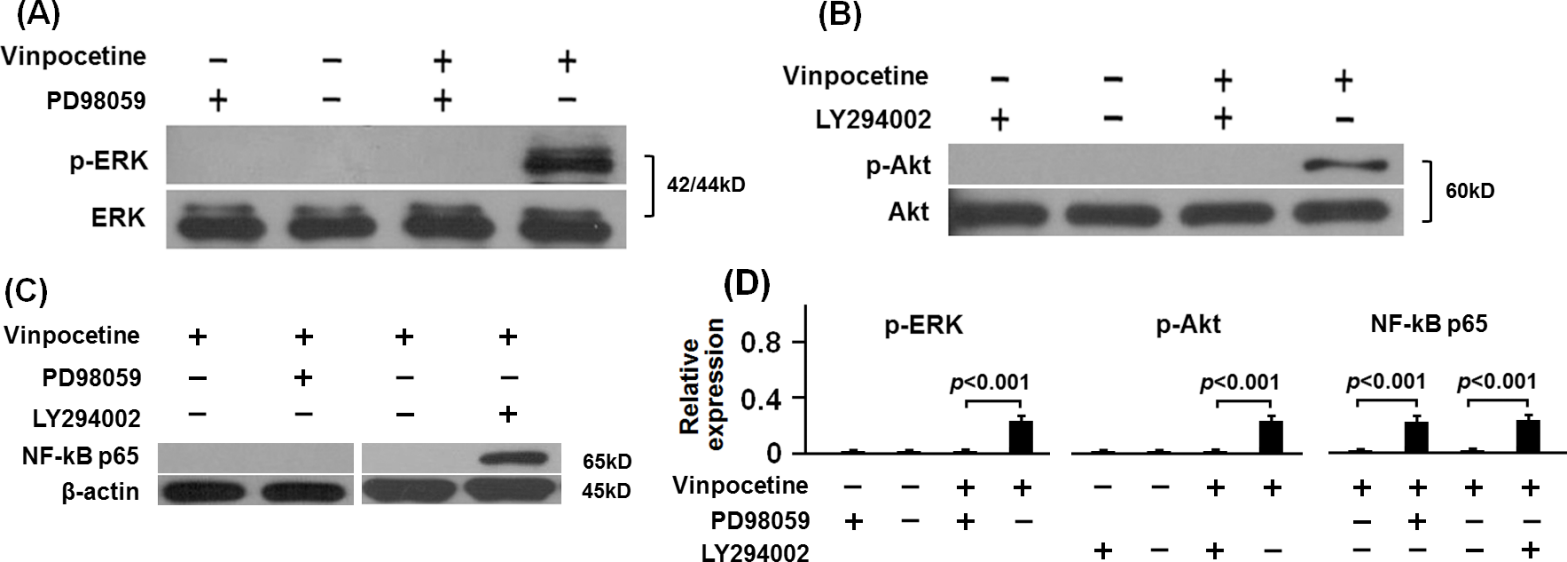
Vinpocetine reduced the translocation of NF-кB p65 via phosphorylation of Akt. PD98059 and LY294002 were respectively added into the culture of β-GP-stimulated VSMCs with or without vinpocetine treatment. The results showed that phosphorylation of both ERK and Akt were blockaded by their inhibitor (A, B and D). Typically, LY294002 significantly blockaded the inhibitory effect of vinpocetine on translocation of NF-кB in β-GP-stimulated VSMCs (C-D). Representative results from three independent experiments were shown.

**Fig 6:**
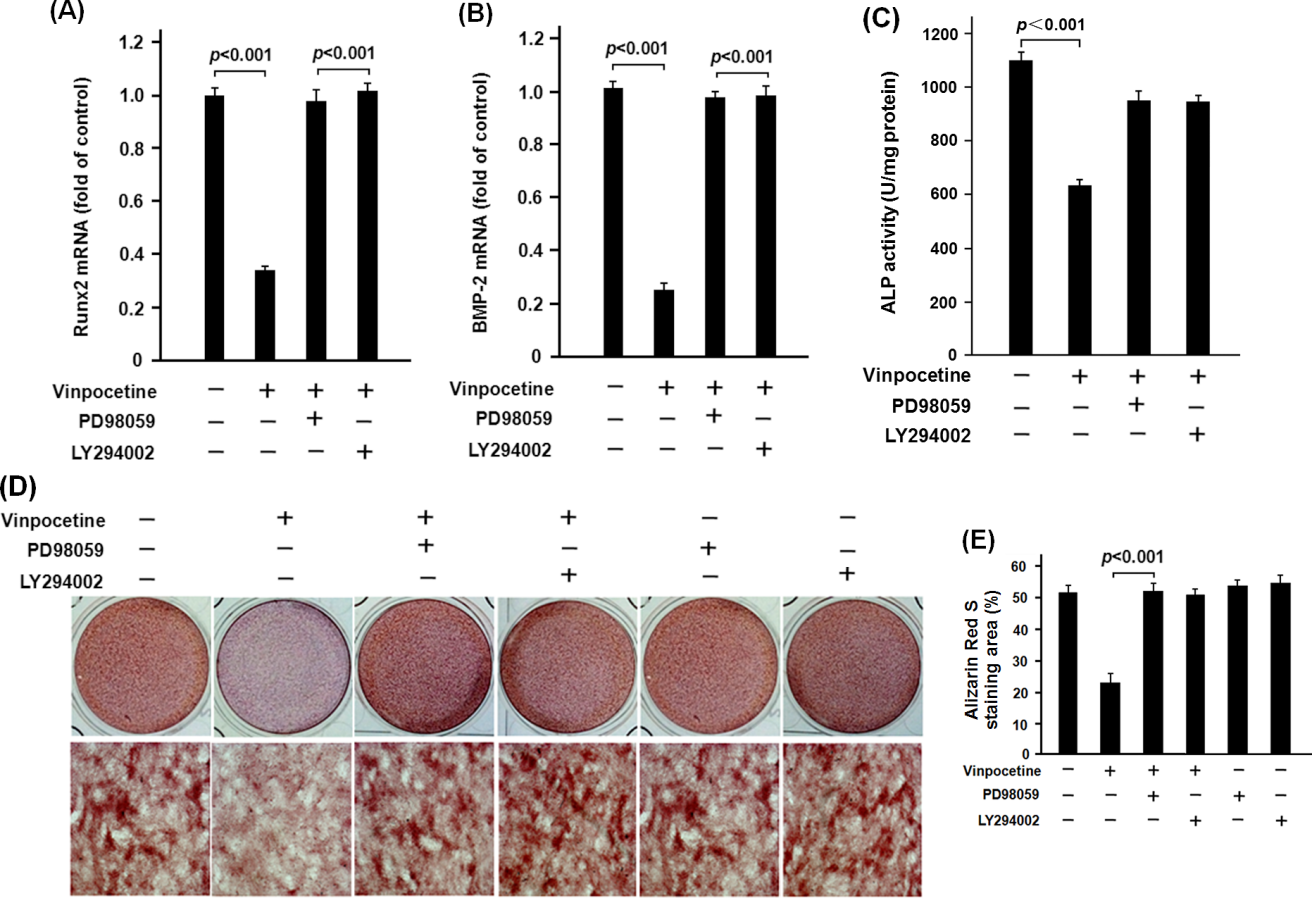
PD98059 and LY294002 attenuated the effect of vinpocetine on osteoblastic differentiation of β-GP-stimulated VSMCs. PD98059 and LY294002 were respectively added into the culture of β-GP-stimulated VSMCs with or without vinpocetine treatment. After a culture of 48h, the cells were harvested for mRNA expression of Runx2 and BMP-2 as well as the ALP activity. Our data showed that both PD98059 and LY294002 significantly inhibited the mRNA expression of Runx2 (A) and BMP-2 (B) as well as the ALP activity (C). After a culture of 18 days, the results of Alizad S staining showed that both PD98059 and LY294002 significantly blockaded the inhibitory effect of vinpocetine on formation of mineralized nodule in calcifying VSMCs. Representative results from three independent experiments were presented.

In Fig 5, PD98059 and LY294002 significantly blockaded the vinpocetine-induced phosphorylation of ERK and Akt respectively. Notably, LY294002 significantly attenuated the inhibitory effect of vinpocetine on NF-кB p65 translocation in β-GP-stimulated VCMCs. To confirm the role of ERK and Akt in action mechanism of vinpocetine on osteoblastic differentiation of VSMCs, mRNA expression of Runx2 and BMP-2 as well as the ALP activity was analyzed. As it was shown in Fig 6A–6C, both PD98059 and LY294002 significantly blockaded the inhibitory effect of vinpocetine on mRNA expression of Runx2 and BMP-2 as well as the ALP activity in β-GP-stimulated VSMCs. The Alizarin Red S staining showed that the inhibitory effect of vinpocetine on the formation of mineralized nodule in β-GP-stimulated VSMCs was significantly blockaded by PD98059 and LY294002 (Fig 6D and 6E).

### Vinpocetine inhibited the osteoblastic differentiation of VSMCs through TSPO

**Fig 7:**
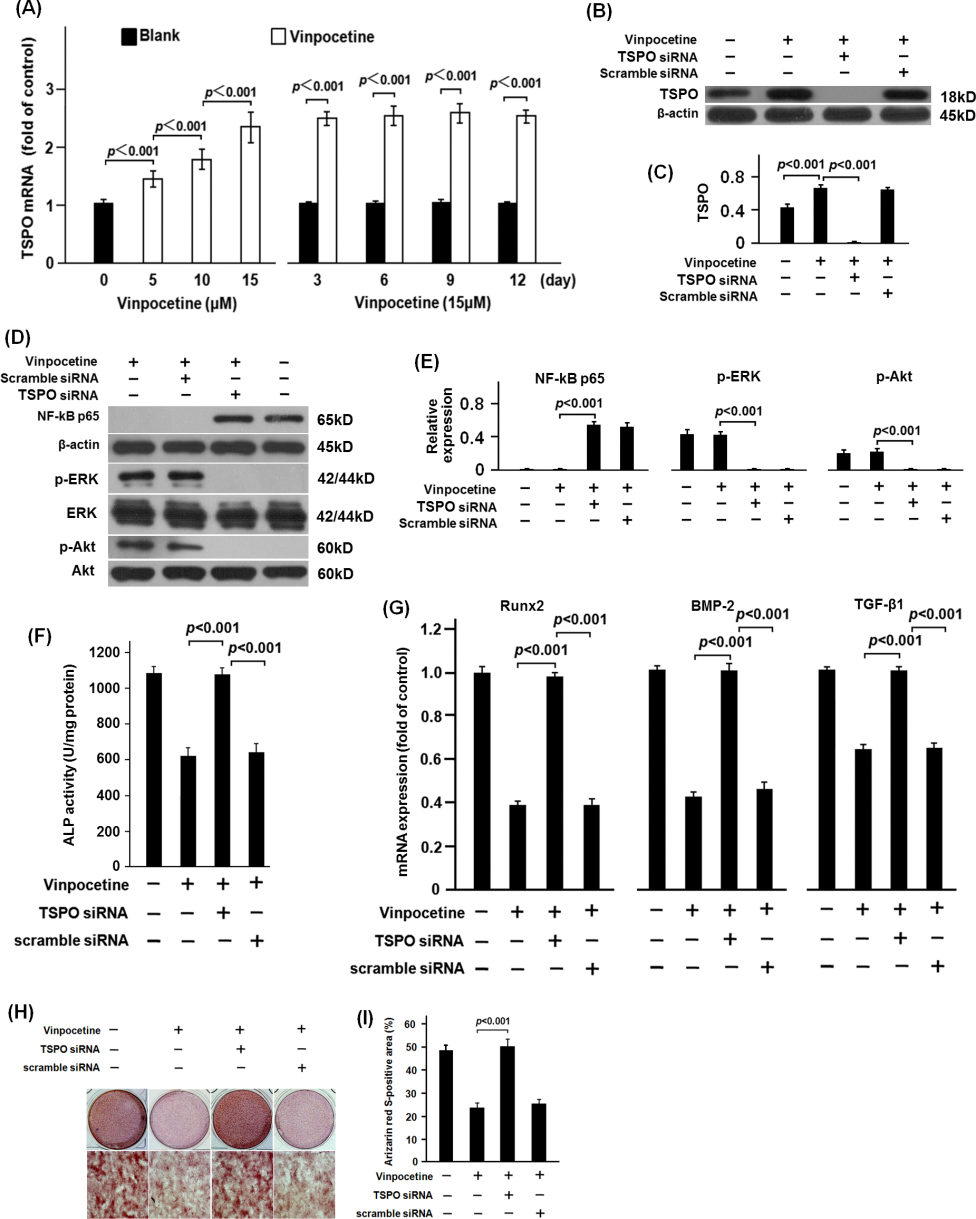
Vinpocetine inhibited the osteoblastic differentiation of VSMCs via TSPO. As it was shown, VSMCs were treated with or without vinpocetine under different conditions. After the culture, cells were harvested for the analysis of the mRNA expression of TSPO. Our data showed that vinpocetine significantly promoted the mRNA expression of TSPO in VSMCs (A). β-GP-stimulated VSMCs were transfected with TSPO siRNA or the scramble siRNA in the presence of 15μM vinpocetine. The Western blot analysis showed that TSPO siRNA significantly silenced the expression of TSPO in VSMCs (B-C). TSPO siRNA significantly blockaded the effect of vinpocetine on phosphorylation of ERK and Akt as well as the translocation of NF-кB p65 in β-GP-stimulated VSMCs (D-E). Meanwhile, the inhibitory effect of vinpocetine on both ALP activity and the mRNA expression of Runx2, BMP-2 and TGF-β1 in β-GP-stimulated VSMCs were significantly blockaded by the TSPO siRNA (F-G). The Alizad S staining showed that TSPO siRNA significantly attenuated the inhibitory effect of vinpocetine on β-GP-induced matrix mineralization of VSMCs (H-I). Representative results from three independent experiments were shown.

TSPO has been demonstrated to be the specific receptor for vinpocetine. In both the dose-gradient and time-gradient experiment, our data showed that 5 to 15 μM of vinpocetine could significantly upregulate the mRNA expression of TSPO (Fig 7A). To explore if vinpocetine inhibits the osteoblastic differentiation of VSMCs via TSPO, siRNA targeting TSPO were used in this study. In Fig 7B and 7C, expression of TSPO in VSMCs was significantly silenced by TSPO siRNA. Furthermore, TSPO siRNA significantly attenuated the effect of vinpocetine on phosphorylation of ERK and Akt as well as the translocation of NF-кB p65 in the β-GP-stimulated VCMCs (Fig 7D and 7E). To further confirm the inhibitory effect of vinpocetine on osteoblastic differentiation of VSMCs via TSPO, ALP activity and mRNA expression of Runx2, BMP-2 and TGF-β1 were analyzed. Our data showed that silencing of TSPO significantly blockaded the inhibitory effect of vinpocetine on ALP activity and mRNA expression of Runx2, BMP-2 and TGF-β1 in β-GP-stimulated VSMCs (Fig 7F and 7G). Finally, the Alizarin Red S staining area in β-GP-stimulated VSMCs treated with vinpocetine was significant reduced by TSPO siRNA (Fig 7H and 7I).

## Discussion

Vascular calcification is an active process similar to osteogenesis, in which the predominant cells, vascular smooth muscle cells acquire the osteogenic phenotype with the increase of ALP activity and expression of osteocalcin, collagen type I, Runx2 and BMP-2 [10,13,15]. Our current study demonstrated that vinpocetine significantly reduced the expression of osteogenic phenotype in β-GP-stimulated VSMCs, which suggests that vinpocetine may be a promising candidate for the clinical therapy of vascular calcification.

Previously, it has been demonstrated that 25–75 μM of vinpocetine for 3 days significantly inhibited the proliferation of VSMCs which was induced by reactive oxidative species production [9]. However, we observed significant apoptosis of VSMCs by 30 μM or more of vinpocetine. In this study, we observed that vinpocetine significantly downregulated expression of Runx2, osteocalcin, collagen type I, BMP-2 as well as ALP activity in β-GP-stimulated VSMCs. The Alizarin Red S staining confirmed that vinpocetine significantly reduced the formation of mineralized nodule in β-GP-stimulated VSMCs. Our data demonstrated that vinpocetine can attenuate the osteoblastic differentiation of VSMCs. Previously, we have demonstrated that both MAPK and PI3K/Akt signaling pathway participate in the modulation of osteoblastic differentiation of VSMCs [10,16]. Given that vinpocetine inhibited the high glucose-induced proliferation of VSMCs via preventing ROS activation and affecting MAPK, PI3K/Akt, and NF-кB signaling [8], we detected the phosphorylation of MAPK and Akt signaling molecules as well as the translocation of NF-кB p65 in β-GP-stimulated VSMCs with or without vinpocetine. As expected, our data showed that both ERK and Akt were significantly phosphorylated by the vinpocetine treatment, especially on the 15 min after the treatment. The expression of NF-кB p65 in the nucleus was opposite to the phosphorylation of ERK and Akt, which suggests that vinpocetine may inhibit the translocation of NF-кB and attenuate the osteoblastic differentiation of VSMCs via ERK/Akt signaling pathway. To further explore the relation between ERK/Akt and NF-кB signaling, we investigated the effect of PD98059 and LY294002 on the translocation of NF-кB p65 in β-GP-stimulated VSMCs with the treatment of vinpocetine. Notably, our result suggested that phosphorylation of Akt mediated the inhibition of vinpocetine on translocation of NF-кB p65 in calcifying VSMCs. Given that both PD98059 and LY294002 significantly increased ALP activity, expression of Runx2 and BMP-2 as well as the formation of mineralized nodule in calcifying VSMCs treated vinpocetine, our results indicated that both ERK and Akt were involved in the action mechanism of vinpocetine on osteoblastic differentiation of VSMCs. Vinpocetine may employ multiple signaling pathways to exert its inhibitory effect on osteoblastic differentiation of VSMCs. Vinpocetine induced the phosphorylation of Akt to inhibit the translocation of NF-кB p65 while the downstream molecule after the phosphorylation of ERK by vinpocetine remains unknown. Further study is necessary to elucidate the ERK signaling pathway in action mechanism of vinpocetine on the osteoblastic differentiation of VSMCs.

TSPO is a specific receptor of vinpocetine and mediates the neuroprotective effect of vinpocetine by inducing the anti-inflammatory effect [11]. To further elucidate the action mechanism of vinpocetine on vascular calcification, we investigated the expression of TSPO on the VSMCs. In dose and time-gradient experiments, vinpocetine significantly upregulated the mRNA expression of TSPO. Relying on the RNA interference, we demonstrated that TSPO was expressed by VSMCs and the designed TSPO siRNA silenced the expression of TSPO efficiently. Despite of the upregulating ALP activity, mRNA expression of Runx2, and BMP-2 as well as the formation of mineralized nodule in calcifying VSMCs treated with vinpocetine, silencing of TSPO also blockaded the inhibitory effect of vinpocetine on mRNA expression of TGF-β1. TGF-β1 binding to its receptor has been demonstrated to play a crucial role in vascular calcification [17]. Our results supports that vinpocetine binding to TSPO inhibits the osteoblastic differentiation of VSMCs induced by β-GP. It has been demonstrated that TSPO is directly or indirectly involved in multiple physiological process including apoptosis, cell proliferation, differentiation and regulation of mitochondrial function [18]. Our study suggested that TSPO also participates in the regulation of osteoblastic differentiation of VSMCs.

## Conclusion

Taken together, our study presented the first evidences that vinpocetine could attenuate the osteoblastic differentiation of VSMCs via inducing the phosphorylation of ERK and Akt, in which the phosphorylation of Akt inhibited the translocation of NF-кB p65. Our data suggested vinpocetine may be a promising strategy for the clinical therapy of vascular calcification. However, further study based on the animal experiment is necessary to confirm the effect of vinpocetine on vascular calcification.
